# Low burden transthyretin cardiac amyloidosis on cardiac magnetic resonance: comprehensive phenotyping and distinction from hypertrophic phenocopies

**DOI:** 10.1093/ehjimp/qyag038

**Published:** 2026-02-28

**Authors:** Bethlehem Mengesha, Suman Prabhakar, Gary R Small, Sharon Chih, Rebecca Thornhill, D Ian Paterson

**Affiliations:** University of Ottawa Heart Institute, University of Ottawa, 40 Ruskin street, Ottawa, Ontario, Canada K1Y 4W7; The Ottawa Hospital, University of Ottawa, 501 Smyth Rd, Ottawa, Ontario K1H 8L6; University of Ottawa Heart Institute, University of Ottawa, 40 Ruskin street, Ottawa, Ontario, Canada K1Y 4W7; The Ottawa Hospital, University of Ottawa, 501 Smyth Rd, Ottawa, Ontario K1H 8L6; University of Ottawa Heart Institute, University of Ottawa, 40 Ruskin street, Ottawa, Ontario, Canada K1Y 4W7; The Ottawa Hospital, University of Ottawa, 501 Smyth Rd, Ottawa, Ontario K1H 8L6; University of Ottawa Heart Institute, University of Ottawa, 40 Ruskin street, Ottawa, Ontario, Canada K1Y 4W7; The Ottawa Hospital, University of Ottawa, 501 Smyth Rd, Ottawa, Ontario K1H 8L6; University of Ottawa Heart Institute, University of Ottawa, 40 Ruskin street, Ottawa, Ontario, Canada K1Y 4W7; The Ottawa Hospital, University of Ottawa, 501 Smyth Rd, Ottawa, Ontario K1H 8L6; University of Ottawa Heart Institute, University of Ottawa, 40 Ruskin street, Ottawa, Ontario, Canada K1Y 4W7; The Ottawa Hospital, University of Ottawa, 501 Smyth Rd, Ottawa, Ontario K1H 8L6

**Keywords:** ATTR cardiac amyloidosis, cardiac MRI, early detection

## Abstract

**Aims:**

Transthyretin cardiac amyloidosis (ATTR CA) is a progressive disease arising from the deposition of amyloid fibrils in the myocardium. Cardiac magnetic resonance (CMR) tissue characterization imaging, including myocardial extracellular volume (ECV) fraction, is used to detect amyloid infiltration, but the identification of early-stage disease is challenging. We sought to describe the phenotype of low burden ATTR CA on CMR and identify imaging features that allow differentiation from potential disease mimickers.

**Methods and results:**

Eighty-three patients with ATTR CA and prior contrast-enhanced CMR were stratified by quartiles of ECV into low (ECV ≤43%) or higher (ECV >43%) burden groups. Global and regional function and myocardial tissue characterization were used to phenotype disease. Receiver operating characteristic analysis was performed to assess the diagnostic performance of CMR for distinguishing low burden ATTR CA from hypertensive heart disease (HHD) and mild hypertrophic cardiomyopathy (HCM). Among 22 patients with low ECV burden, CMR measures of amyloid infiltration predominantly affected the basal left ventricular (LV) segments with progressive involvement of the mid and apical regions at higher ECV. Global myocardial late gadolinium enhancement (LGE) and ECV showed high accuracy for differentiating low burden ATTR CA from HHD and mild HCM, area under the curve (AUC) of 0.99 and 0.97, respectively, compared to strain-based measures, AUC 0.47–0.82.

**Conclusion:**

Tissue characterization imaging (myocardial ECV and LV LGE) can be used to distinguish low burden ATTR from potential disease mimickers and appears to outperform traditional strain-based measures.

## Graphical Abstract

**Graphical Abstract:**
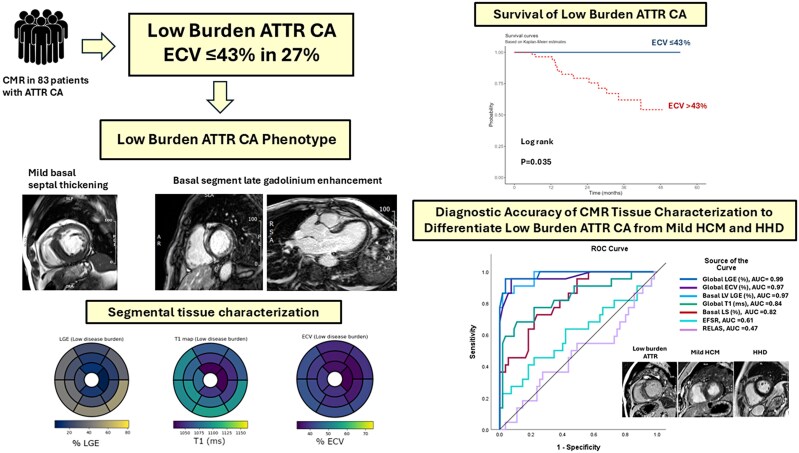
The figure summarizes key study findings, including cardiac magnetic resonance imaging characteristics of patients with low burden ATTR CA (left panel). The lower event rates in patients with low burden ATTR cardiac amyloidosis compared to higher burden disease (right upper panel). Receiver operator characteristics curve of CMR parameters differentiating low burden ATTR cardiac amyloidosis from potential mimickers (right lower panel).

## Introduction

Amyloidosis is a progressive and fatal systemic disease characterized by deposition of amyloid fibrils in vital organs, including the heart. While more than 30 precursor amyloidogenic proteins have been identified, the majority of patients with cardiac involvement are affected by transthyretin (ATTR) amyloidosis.^1–3^ ATTR cardiomyopathy is increasingly recognized in the elderly and has been described in 12–13% of patients with heart failure with preserved ejection fraction, 5–7% of patients with suspected hypertrophic cardiomyopathy, and 8% of those with severe aortic stenosis referred for transcatheter aortic valve implantation.^2,4^

Diagnostic workflows for patients with suspected ATTR cardiac amyloidosis (CA) include scintigraphy with bone-avid tracers as a first-line investigation, given high sensitivity and specificity for this subtype.^5,6,7^ Cardiac magnetic resonance (CMR) is also sensitive to the detection of CA but does not reliably distinguish ATTR from other amyloidosis subtypes. However, CMR has the advantage of characterizing myocardial tissue through the quantification of extracellular volume (ECV). This is particularly relevant in the context of CA, where the expansion of ECV in response to amyloid deposition in the extracellular space can serve as a surrogate for quantifying disease burden.^3,6^ In addition, myocardial ECV ≥59% is associated with increased mortality in ATTR CA.^8^

In clinical practice, ATTR disease staging, such as proposed by Gillmore *et al*., utilizes circulating biomarkers b-type natriuretic peptide (NT-pro-BNP) and estimated glomerular filtration rate to evaluate disease burden, with early-stage disease having better outcomes than later stages.^9^ These measures may also predict response to therapies. A recent subgroup analysis of the HELIOS-B trial found that patients with NT-proBNP > 2000pg/mL were unlikely to derive benefit from ATTR targeting drug therapy.^10^ To date, cardiac imaging parameters have not been included in ATTR disease staging algorithms.

Furthermore, no studies have attempted to describe early-stage disease on cardiac imaging. CMR is ideally positioned to assist in the early diagnosis of CA in view of the extensive tissue characterization available with this modality. In addition, CMR is a recommended test in undifferentiated cardiomyopathies following initial echocardiography.^7^ However, the diagnostic performance of CMR for ATTR CA in this setting is not well established. We sought, therefore, to characterize the morphologic and multiparametric features of low burden ATTR-CA on CMR and compare these findings to patients with higher burden ATTR CA and those with similar phenotypes.

## Methods

### Patient populations

This is a retrospective study of consecutive patients undergoing contrast-enhanced CMR at 1.5T for suspected CA between 2017 and 2024 at a tertiary care hospital (University of Ottawa Heart Institute, Ontario, Canada). The study protocol was approved by our institutional ethics review board. We initially identified 171 eligible patients, but excluded 28 without a definitive diagnosis of CA and 35 with light chain-associated cardiac amyloidosis. We further excluded 25 patients with non-contrast CMR studies, which left 83 patients for inclusion. Patients’ demographic and clinical data at baseline and follow-up were collected from electronic medical records (EPIC, Wisconsin, USA). All patients had a confirmed diagnosis of ATTR CA with grade 2–3 tracer uptake on pyrophosphate scintigraphy or based on a positive endomyocardial biopsy with Congo red staining and ATTR identified on mass spectroscopy.

Consecutive patients with hypertensive heart disease (HHD) or hypertrophic cardiomyopathy (HCM) referred for CMR during the study period to evaluate unexplained LV hypertrophy were included as comparator groups for patients with low burden ATTR CA. HCM was defined as LV wall thickness of ≥15 mm or more limited hypertrophy (13–14 mm) with evidence of a hereditary cause.^11^ We excluded patients with unconfirmed HCM, apical HCM, LV dysfunction, prior myectomy, alcohol septal ablation, or prior myocardial infarction and absence of tissue mapping or gadolinium contrast administration on CMR. The cohort was further restricted to patients with mild HCM, which was defined as maximal wall thickness ≤17 mm, to approximate wall thickness in the low burden ATTR CA group.

Hypertensive heart disease was defined by the presence of LV wall thickness >12 mm on CMR and an associated diagnosis of systemic hypertension in the absence of significant left-sided valvular heart disease, chronic kidney disease, or underlying cardiomyopathy.

### CMR protocol and image analysis

All patients underwent a contrast-enhanced CMR examination on a 1.5 T scanner (Magnetom Aera, Siemens Healthineers, Erlangen, Germany) with image acquisition according to recommended protocols for patients with non-ischaemic cardiomyopathy.^12^ We acquired standard cine imaging for cardiac structure and function assessment and performed myocardial tissue characterization with late gadolinium enhancement (LGE) imaging, native T1 mapping, and extracellular volume fraction. Images were analysed on commercial software (CVI42 Version 6.1, Circle Inc, Calgary, AB, Canada) by an experienced reader blinded to group assignment and following established guidelines for post-processing^13^ (see Supplementary data online, *Appendix* for additional details on the CMR protocol). The upper limit of normal wall thickness was defined as 12 mm for the LV and 5 mm for the RV.^14,15^ Relative apical sparing ratio (RELAS) was defined as a ratio of longitudinal strain (LS) at the apex divided by the mean of LS at mid-ventricle and base, and the ejection fraction strain ratio (EFSR) was defined as LVEF divided by global LS.^16^

The extent of LGE was quantified by using full width half maximum technique and was expressed segmentally using a 16-segment model^17^ and globally as % of total LV mass. Patients were further divided into low, moderate, and high disease burden based on ECV quartiles. Patients in the first quartile (ECV 28–43%) represented a low burden of ATTR CA, whereas those in the second and third quartile (ECV 43.1–56.5%) were combined to define patients with moderate disease burden, and those in the fourth quartile (ECV 56.6–73%) defined high disease burden. To assess inter-reader reproducibility, a second experienced CMR reader (SP) reevaluated T1, ECV, and LGE measures in a subset of cases.

### Statistics

Statistical analysis was performed using SPSS (IBM SPSS Statistics 29.0.2.0) and Jamovi project 2.3.28. Categorical variables were reported as frequencies and percentages. Continuous variables were reported in mean and standard deviation (SD) or median and interquartile range, as appropriate. Distribution of continuous variables was tested using Shapiro–Wilk test. Categorical variables were compared using chi-square and Fisher's exact tests, as appropriate. Differences between group means, or medians among disease amyloid disease strata were assessed using one-way ANOVA or Kruskal–Wallis tests, as appropriate. Variables demonstrating significant group effects on ANOVA or Kruskal–Wallis tests underwent *post hoc* Games–Howell test or Dwass-Steel-Crichtlow-Fligner pairwise comparison, as appropriate. Intraclass correlation coefficients (ICC) were calculated to assess the reproducibility of global and segmental T1 and ECV estimates. Kappa statistics were used to assess inter-reader agreement regarding the presence of atrial and RV LGE. Receiver operating characteristic (ROC) curves were constructed to assess the performance of each of the following imaging parameters in differentiating low burden ATTR from mild HCM and HHD. LV LGE %, the presence of atrial and RV LGE, and a combined mean LGE% of basal inferior and lateral segments. Youden’s index was used to determine the optimal threshold for discriminating low burden ATTR CA from mild HCM and HHD. Survival analysis was evaluated using Kaplan–Meier curve and groups compared with a log-rank test.

## Results

Eighty-three patients with confirmed ATTR CA (mean age 79 ± 9, males 87%) and prior CMR during the study period were included. The diagnosis of ATTR CA was confirmed with a pyrophosphate scan in 80 (96%) and an endomyocardial biopsy in 3 (4%). Nine (14%) patients had hereditary ATTR, including 7 with the Val 142lle mutation. Asymmetric septal LV wall thickening was the most common morphology, 66 (80%), whereas 10 (12%) had normal wall thickness, and 7 (8%) had symmetrical wall thickening (see Supplementary data online, *Figure S1*).

Gillmore staging of ATTR CA in our study cohort identified 45 (55%) patients with stage 1, 28 (34%) with stage 2, and 9 (11%) with stage 3 disease (see Supplementary data online, *Table S1*). There was no significant difference in global myocardial ECV on CMR according to Gillmore stage, mean ECV 49 ± 11% for stage 1, ECV 53 ± 9% for stage 2 and 53 ± 8% for stage 3, log rank *P* = 0.225 (see Supplementary data online, *Table S2*).

### Clinical characteristics of low burden ATTR CA

When stratifying the study group using ECV as a surrogate for disease burden, 22 patients had low disease burden (ECV Q1 28–43%), 40 patients had moderate disease burden (ECV Q2 43.1–51% and Q3 51.1–56.5%) and 21 patients had high disease burden (ECV Q4 56.6–73%) (*Table 1*). Pseudo-infarct pattern on electrocardiogram was more common in moderate and high burden patients (63% and 45%, respectively) compared to low (5%), *P* < 0.001. Cardiac biomarkers were elevated in the low burden group (median NT-pro BNP 552 ng/L and median troponin T 36 ng/L) but were significantly lower than the moderate and high burden groups, *P* = 0.002 for comparison with NT-pro BNP and *P* = 0.004 for comparison with troponin T (*Table 1*).

**Table 1:** Demographics and clinical parameters of patients with ATTR cardiac amyloidosis according to extracellular volume burden

Clinical variables	Low burden [ECV 28–43%] *n* = 22	Moderate burden [ECV 43.1–56.5%] *n* = 40	High burden [ECV 56.6–73%] *n* = 21	*P* value
Age	76 ± 9	80 ± 8	79 ± 10	0.120
Male sex	16 (73%)	36 (90%)	20 (95%)	0.066
Variant ATTR	5 (23%)	2 (5%)	1 (5%)	0.053
Body mass index (kg/m^2^)	26 ± 5	26 ± 4	25 ± 3	0.516
Systolic BP (mmHg)	136 ± 23	129 ± 17	121 ± 13^b^	**0.021**
Diastolic BP (mmHg)	75 ± 12	75 ± 13	78 ± 16	0.743
Heart rate (/min)	82 ± 17	70 ± 13^a^	80 ± 19	**0.013**
Hypertension	16 (73%)	27 (60%)	8 (38%)	**0.036**
Diabetes mellitus	4 (18%)	6 (15%)	3 (14%)	0.928
Atrial fibrillation	6 (27%)	24 (60%)	9 (43%)	**0.043**
Coronary artery disease	6 (27%)	7 (18%)	8 (38%)	0.207
Congestive heart failure				**0.002**
HFrEF	3 (14%)	3 (8%)	11 (53%)	
HFmrEF	6 (27%)	19 (48%)	5 (24%)	
HFpEF	5 (23%)	5 (13%)	1 (5%)	
NYHA class				0.394
Class I	10 (45%)	13 (33%)	3 (14%)	
Class II	7 (32%)	16 (40%)	12 (57%)	
Class III/IV	5 (23%)	11 (28%)	6 (29%)	
Diuretics	8 (36%)	21 (53%)	15 (71%)	0.070
Furosemide dose (mg)	0 (0–20)	20 (0–40)	40 (0–60)^b^	**0.022**
Tafamidis	11 (50%)	25 (63%)	17 (81%)	0.104
Low voltage	3 (14%)	20 (50%)	7 (35%)	**0.023**
Pseudo-infarct pattern	1 (5%)	25 (63%)	9 (45%)	**<0.001**
NT-pro BNP (ng/L)	552 (158–2654)	2740 (1337–5016)^a^	2810 (1540–8496)^b^	**0.002**
Troponin T (ng/L)	36 (26–49)	56 (40–89)^a^	65 (41–82)^b^	**0.004**
eGFR (mL/m/1.73 m sq)	66 (49–84)	66 (56–77)	58 (46–77)	0.343
Gillmore stage (*n* = 82)				0.502
Stage 1	15 (71%)	20 (50%)	10 (48%)	
Stage 2	5 (24%)	15 (38%)	8 (38%)	
Stage 3	1 (5%)	5 (13%)	3 (14%)	

Continuous data is provided as mean ± standard deviation or median and interquartile ranges and categorical data is provided as counts and percentage. *P* < 0.05 for between group comparisons are indicated in bold.

^a^ *P* < 0.05 between low and moderate burden cohorts.

^b^ *P* < 0.05 between low and high burden cohorts.

Abbreviations: ATTR = transthyretin amyloidosis, BP = blood pressure, HFrEF = heart failure with reduced ejection fraction, HFmrEF = heart failure with mildly reduced ejection fraction, HFpEF = heart failure with preserved ejection fraction, NYHA class = New York Heart Association class, BNP = B-type natriuretic peptide, eGFR = estimated glomerular filtration rate (estimated using CKD-EPI formula).

### CMR phenotype of low burden ATTR CA

In the low burden ATTR CA group, 8 patients (36%) had normal LV wall thickness, compared to 2 patients in the moderate and none in the high burden groups, *P* < 0.001, and LV mass was lower in patients with low burden disease (*Table 2*). Only 5% of patients with low burden ATTR CA had increased right ventricular (RV) wall thickness compared to 50% with moderate and 71% with high burden (*P* < 0.001). Left ventricular function was borderline reduced, with a mean LV ejection fraction (EF) 52 ± 12%, and RV function was preserved in the low burden group, whereas LV strain was abnormal (*Table 2*).

**Table 2:** CMR findings in patients with ATTR cardiac amyloidosis according to extracellular volume burden

Imaging modality parameter	Low burden [ECV 28–43%] *n* = 22	Moderate burden [ECV 43.1–56.5%] *n* = 40	High burden [ECV 56.6–73%] *n* = 21	*P* value
LVEF (%)	52 ± 12	50 ± 10	36 ± 14^b,c^	**<0**.**001**
LVEDVi (mL/m^2^)	82 ± 21	81 ± 15	98 ± 23^c^	**0.016**
LVESVi (mL/m^2^)	36 (27–50)	38 (33–48)	56 (44–80) ^b,c^	**<0.001**
Maximal wall thickness	13.5 ± 3	16 ± 3^a^	18 ± 3^b,c^	**<0.001**
LV massi (g/m^2^)	71 ± 17	81 ± 17	99 ± 16^b,c^	**<0.001**
RVEF (%)	52 ± 13	48 ± 11	39 ± 13^b,c^	**0.001**
RVEDVi (mL/m^2^)	77 ± 14	85 ± 22	93 ± 21^b^	**0.044**
RVESVi (mL/m^2^)	34 (26–45)	38 (33–58)	55 (39–80) ^b,c^	**0.001**
LAVi (mL/m^2^)	55 ± 26	61 ± 14	61 ± 13	0.323
RAVi (mL/m^2^)	46 ± 19	64 ± 27	65 ± 23^b,c^	**0.013**
GLS (%)	−16 ± 3	−13 ± 2^a^	−10 ± 2^b,c^	**<0.001**
GRS (%)	23 ± 8	20 ± 6	15 ± 6^b,c^	**<0.001**
GCS (%)	−15 ± 4	−12 ± 6	−10 ± 3^b^	**<0.019**
RELAS	0.40 ± 0.10	0.51 ± 0.13^a^	0.57 ± 0.19^b^	**<0.001**
Global T1 (ms)	1069 ± 38	1107 ± 45^a^	1126 ± 41^b^	**<0.001**
Basal T1	1091 ± 56	1120 ± 54	1136 ± 44	
Mid T1	1061 ± 44	1097 ± 50	1118 ± 44	
Apical T1	1038 ± 46	1093 ± 52	1117 ± 51	
Global ECV (%)	39 ± 4	51 ± 4^a^	64 ± 5^b,c^	**<0.001**
Basal ECV	42 ± 6	54 ± 5	69 ± 6	
Mid ECV	37 ± 3	49 ± 7	61 ± 4	
Apical ECV	37 ± 5	49 ± 7	59 ± 6	
LGE % of LV mass	29 ± 15	40 ± 13^a^	56 ± 15^b,c^	**<0.001**
Right ventricular LGE (N)	13 (59%)	28 (70%)	20 (95%)	**0.021**
Atrial LGE	20 (91%)	40 (100%)	21 (100%)	0.058
Pericardial effusion	9 (41%)	15 (36%)	8 (38%)	0.879

Continuous data is provided as mean ± standard deviation or median and interquartile ranges and categorical data is provided as counts and percentage. *P* < 0.05 for between group comparisons are indicated in bold.

^a^ *P* < 0.05 between low and moderate burden groups.

^b^ *P* < 0.05 between low and high burden groups.

^c^ *P* < 0.05 between moderate and high burden groups.

Abbreviations: LV = left ventricular, RV = right ventricular, EF = ejection fraction, EDVi = end diastolic volume index, ESVi = end systolic volume, LAVi = left atrial volume index, RAVi = right atrial volume index, GLS = global longitudinal strain, GRS = global radial strain, GCS = global circumferential strain, RELAS = Relative apical sparing ratio, LGE = late gadolinium enhancement. N.B. index = indexed to body surface area.

**Figure 1:**
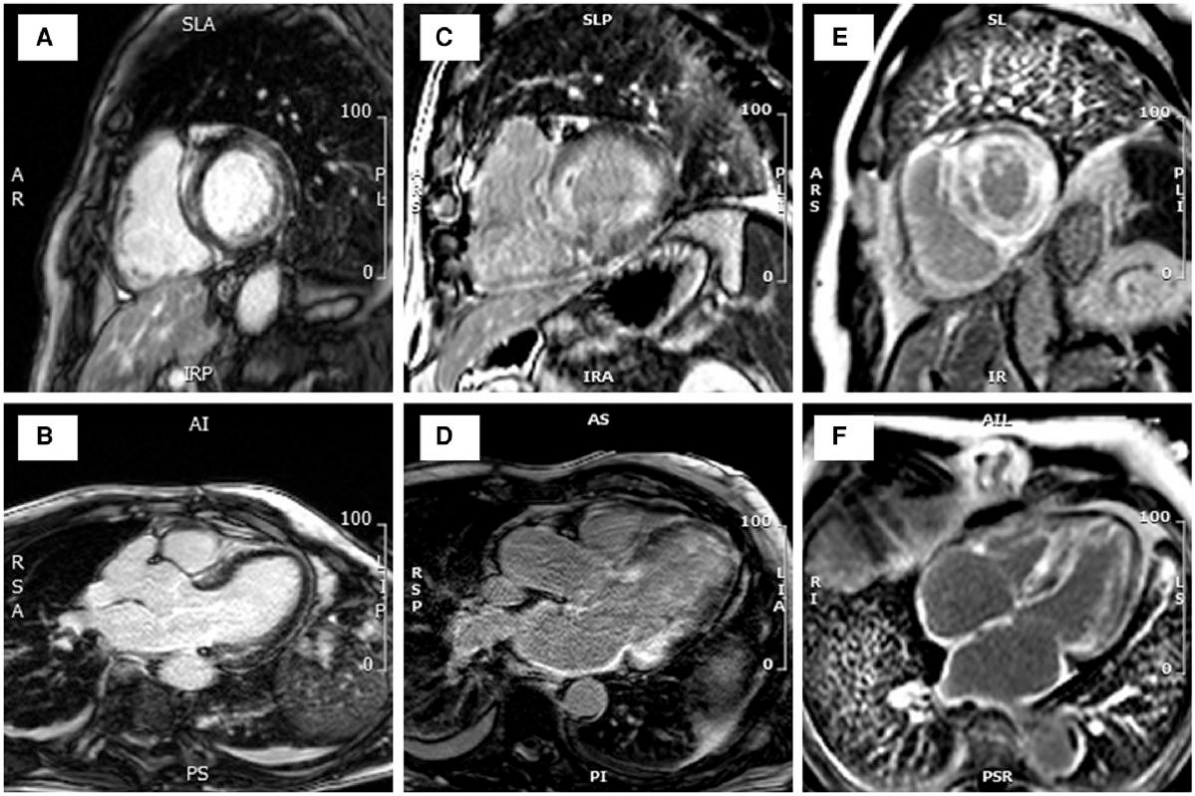
Representative examples of late gadolinium enhancement extent in patients with low burden (panels *A*, *B*), moderate burden (panels *C*, *D*), and high burden (*E*, *F*) ATTR cardiac amyloidosis.

**Figure 2:**
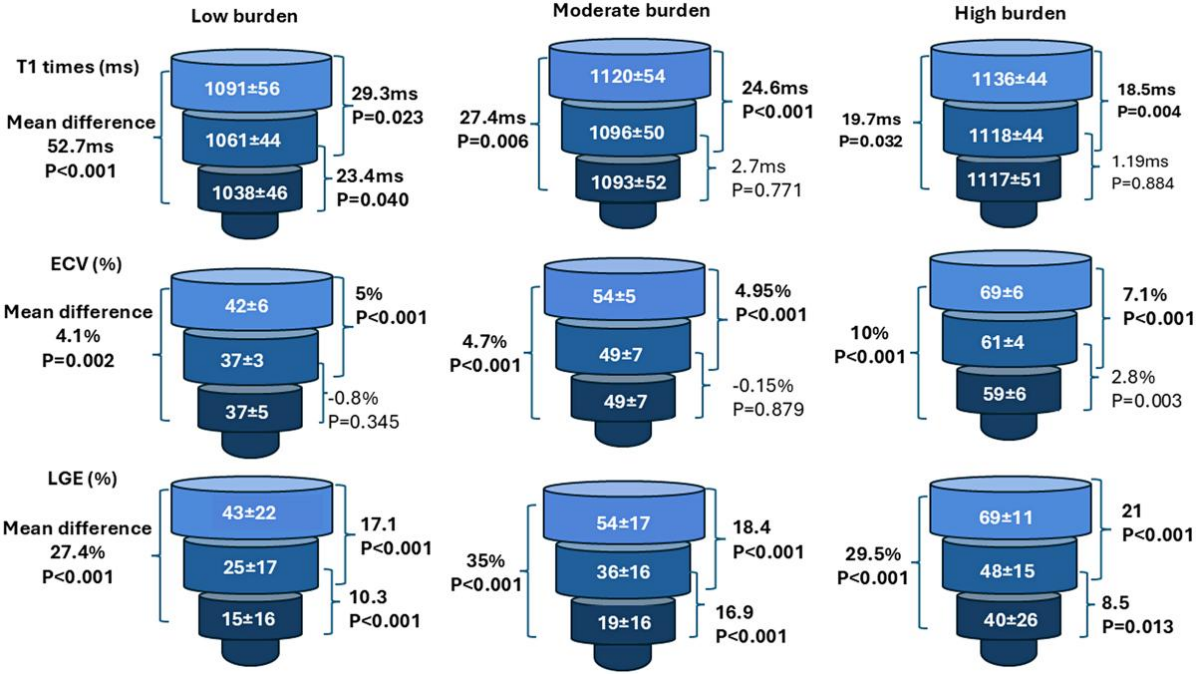
Basal to apical gradients in left ventricular tissue characterization across ATTR CA ECV Strata. Mean +/-SD T1 times, ECV, and LGE % of the basal, mid, and apical segments in low, moderate, and high disease burden. Normal reference values for myocardial T1 = 1000 ± 18 ms, ECV =28 ± 1.5% and LGE 0%.

**Figure 3:**
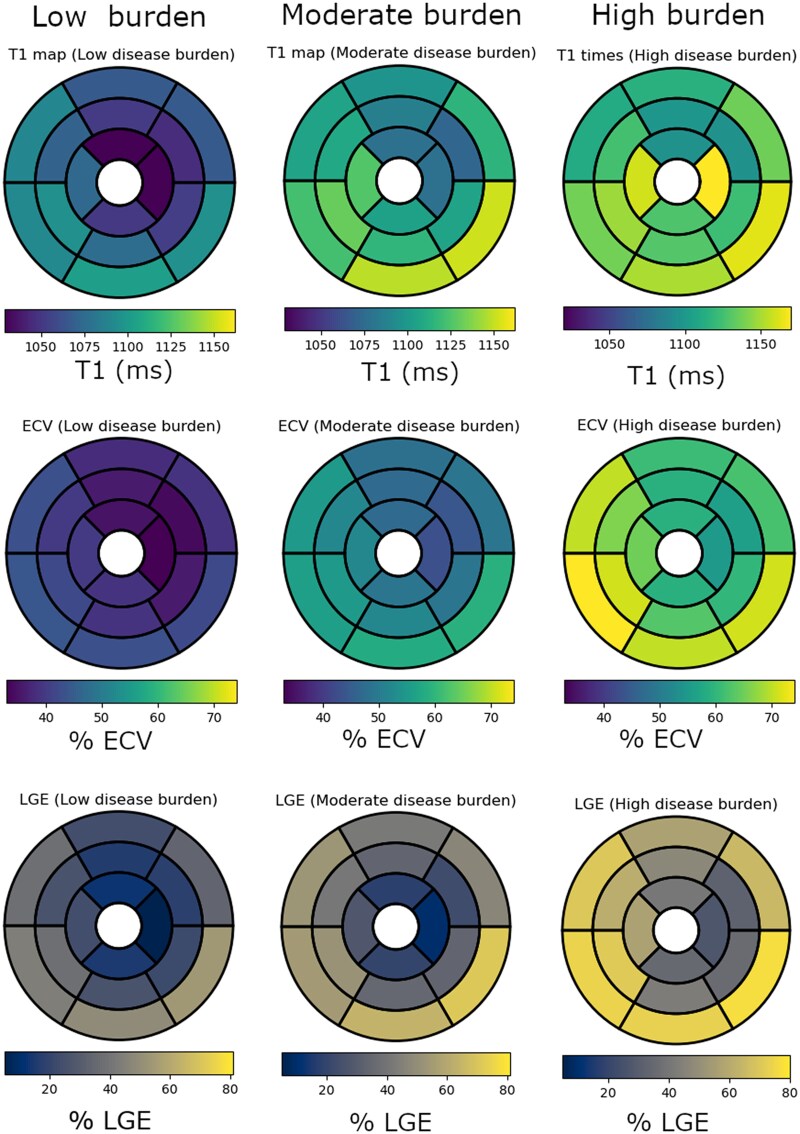
Segmental analysis of tissue characterization across ATTR CA ECV strata. Color map regional representation of mean T1 times, ECV, and LGE% on a 16-segment model, in low, moderate, and high disease burden. Normal reference values for T1 = 1000 ± 18 ms, ECV =28 ± 1.5% and LGE 0%.

Global LV LGE % increased with higher burden, mean LGE 29 ± 15%, 40 ± 13% and 56 ± 15% in the low, moderate, and high burden groups, respectively, *P* < 0.001 (*Figure 1*). Global myocardial T1 in patients with low burden was lower (mean T1 1069 ± 38 ms) compared to moderate and high (mean T1 1107 ± 45 ms, and 1126 ± 41 ms, respectively), *P* < 0.001. The mean T1 times and ECV values were significantly greater in the basal segments compared to apical segments, *P* < 0.001 for each comparison across disease burden strata (*Figure 2*). When T1, ECV values, and LGE % were evaluated using a 16-segment model, low burden ATTR CA is characterized by involvement of the basal inferolateral and adjacent segments and relative sparing of the basal anterior and the mid and apical segments (*Figure 3*).

Inter-reader agreement was excellent with ICCs of 0.96–0.99 for global and 0.83–0.99 for segmental tissue mapping (see Supplementary data online, *Appendix*). Cohen’s kappa agreement between the two readers for atrial wall and RV enhancement was 0.96 and 0.65, respectively.

**Figure 4:**
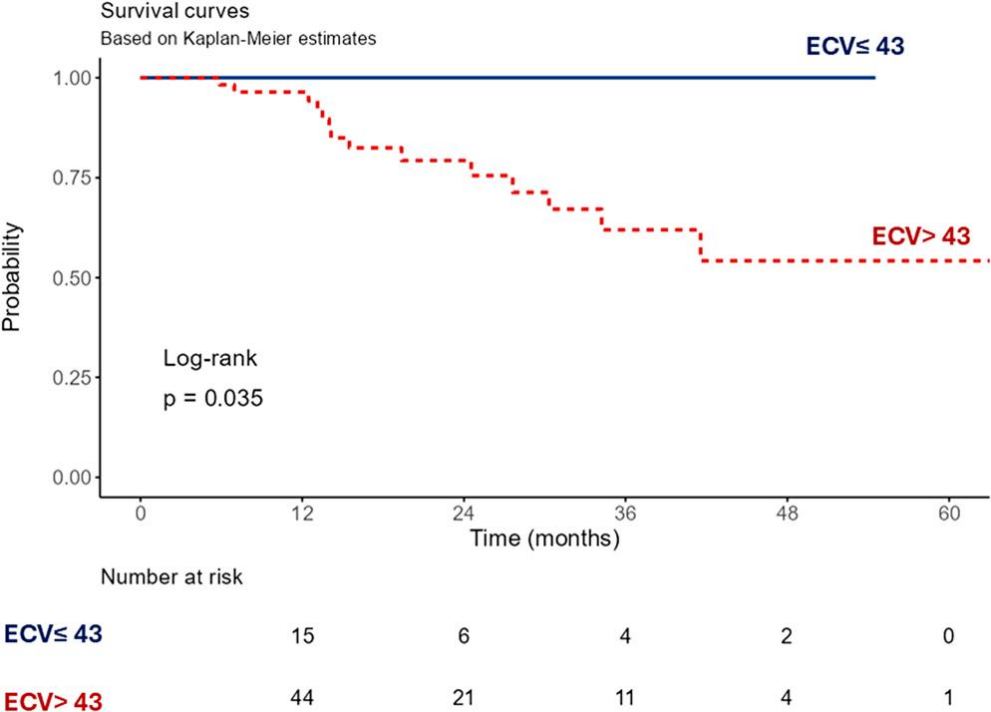
Unadjusted Kaplan–Meier survival curve of patients with ATTR cardiac amyloidosis stratified by myocardial ECV.

During a median follow-up of 1.4 (0.2–7.2) years, 14 patients with ATTR CA died, including 5 (24%) with high disease burden, 9 (23%) with moderate disease burden, and none with low disease burden. Patients with low burden ATTR CA had more favourable survival compared to moderate disease burden and high disease burden on unadjusted Kaplan Meier analysis (*P* = 0.035) (*Figure 4*).

### CMR comparison of low burden ATTR CA with similar hypertrophic phenocopies

The 22 patients with low burden ATTR CA were compared to 27 patients with mild HCM and 28 with HHD. Patients’ baseline characteristics are shown in Supplementary data online, *Table S3*.

On CMR, asymmetric septal hypertrophy was noted in all three groups with a septal to lateral wall ratio 1.8 ± 0.5 in low burden ATTR, 2.3 ± 0.6 in mild HCM, and 2.0 ± 0.7 in HHD, *P* = 0.044 (*Table 3*). Patients with low burden ATTR CA had lower LVEF and RVEF compared to mild HCM and HHD, LVEF 52 ± 12% vs. 65 ± 7% and 63 ± 7% respectively, *P* < 0.001, and RVEF 52 ± 13% vs. 61 ± 7% and 60 ± 7% respectively, *P* = 0.023. Strain analysis also showed impaired global and regional longitudinal strain, circumferential strain, and radial strain in low burden ATTR CA compared to mild HCM and HHD (*Table 3* and Supplementary data online, *Figure S2*). Relative apical sparing of radial and circumferential strain was appreciated in low burden ATTR CA compared to mild HCM and HHD (see Supplementary data online, *Figure S3*).

**Table 3:** Comparison of CMR findings in low burden ATTR cardiac amyloidosis, mild hypertrophic cardiomyopathy, and hypertensive heart disease

Imaging parameters	Low burden ATTR CA *n* = 22	Mild HCM *n* = 27	HHD *n* = 28	*P* value
LVEF (%)	52 ± 12	65 ± 7^a^	63 ± 7^b^	**<0**.**001**
LVEDVi (mL/m^2^)	82 ± 21	72 ± 16	71 ± 17	0.065
LVESVi (mL/m^2^)	36 (27–50)	25 (20–29)^a^	26 (20–33)^b^	**0**.**001**
LV mass index (g/m^2^)	71 ± 17	62 ± 12	63 ± 14	0.075
Maximal wall thickness (mm)	13.5 ± 3	16 ± 1^a^	14 ± 1^c^	**<0**.**001**
Contralateral wall (mm)	8 ± 3	8 ± 2	8 ± 2	0.550
Wall thickness ratio	1.8 ± 0.5	2.3 ± 0.6^a^	2.0 ± 0.7	**0**.**044**
RVEF (%)	52 ± 13	61 ± 7^a^	60 ± 7^b^	**0**.**023**
RVEDVi (mL/m^2^)	77 ± 14	69 ± 12	69 ± 14	0.064
RVESVi (mL/m^2^)	34 (26–45)	25 (21–32)^a^	29 (20–33)	**0**.**022**
LAVi (mL/m^2^)	55 ± 26	50 ± 18	43 ± 15	0.149
LAEF (%)	34 ± 14	53 ± 11	54 ± 14^b^	**0**.**048**
RAVi (mL/m^2^)	46 ± 19	38 ± 12	36 ± 11^b^	**0**.**033**
RAEF (%)	45 ± 17	57 ± 13^a^	55 ± 12^b^	**0**.**016**
GLS (%)	−16 ± 3	−18 ± 2^a^	−18 ± 2^b^	**<0**.**001**
GRS (%)	23 ± 8	34 ± 6^a^	33 ± 8^b^	**<0**.**001**
GCS (%)	−15 ± 4	−19 ± 2^a^	−19 ± 3^b^	**<0**.**001**
RELAS	0.40 ± 0.10	0.41 ± 0.10	0.41 ± 0.10	**0**.**948**
Global T1 (ms)	1069 ± 38	1028 ± 31^a^	1014 ± 27^b^	**<0**.**001**
Basal T1	1091 ± 56	1048 ± 37	1032 ± 26	
Mid T1	1061 ± 44	1020 ± 31	1009 ± 38	
Apical T1	1038 ± 46	997 ± 39	985 ± 47	
Global ECV (%)	39 ± 4	29 ± 3^a^	27 ± 2^b^	**<0**.**001**
Basal ECV	42 ± 6	28 ± 3	29 ± 9	
Mid ECV	37 ± 3	29 ± 4	27 ± 3	
Apical ECV	37 ± 5	31 ± 4	29 ± 3	
LGE % of LV mass	29 ± 15	4 ± 3^a^	1.3 ± 2^b,c^	**<0**.**001**
RV LGE	13 (59%)	0^a^	0^b^	**<0**.**001**
Atrial LGE	20 (91%)	1 (4%)^a^	2 (7%)^b^	**<0**.**001**

Continuous data is provided as mean ± standard deviation or median and interquartile ranges and categorical data is provided as counts and percentage. *P* < 0.05 for between group comparisons are indicated in bold.

^a^ *P* < 0.05 between low burden ATTR and HCM.

^b^ *P* < 0.05 between low burden ATTR and HHD.

^c^ *P* < 0.05 between HCM and HHD.

Abbreviations: ATTR CA = transthyretin cardiac amyloidosis, HCM = hypertrophic cardiomyopathy, HHD = hypertensive heart disease, LV = left ventricular, RV = right ventricular, EF = ejection fraction, EDVi = end diastolic volume index, ESVi = end systolic volume index, SVi = stroke volume index, LAVi = left atrial volume index, RAVi = right atrial volume index, GLS = global longitudinal strain, GRS = global radial strain, GCS = global circumferential strain, RELAS = Relative apical sparing ratio, LGE = late gadolinium enhancement. N.B. index = indexed to body surface area.

**Figure 5:**
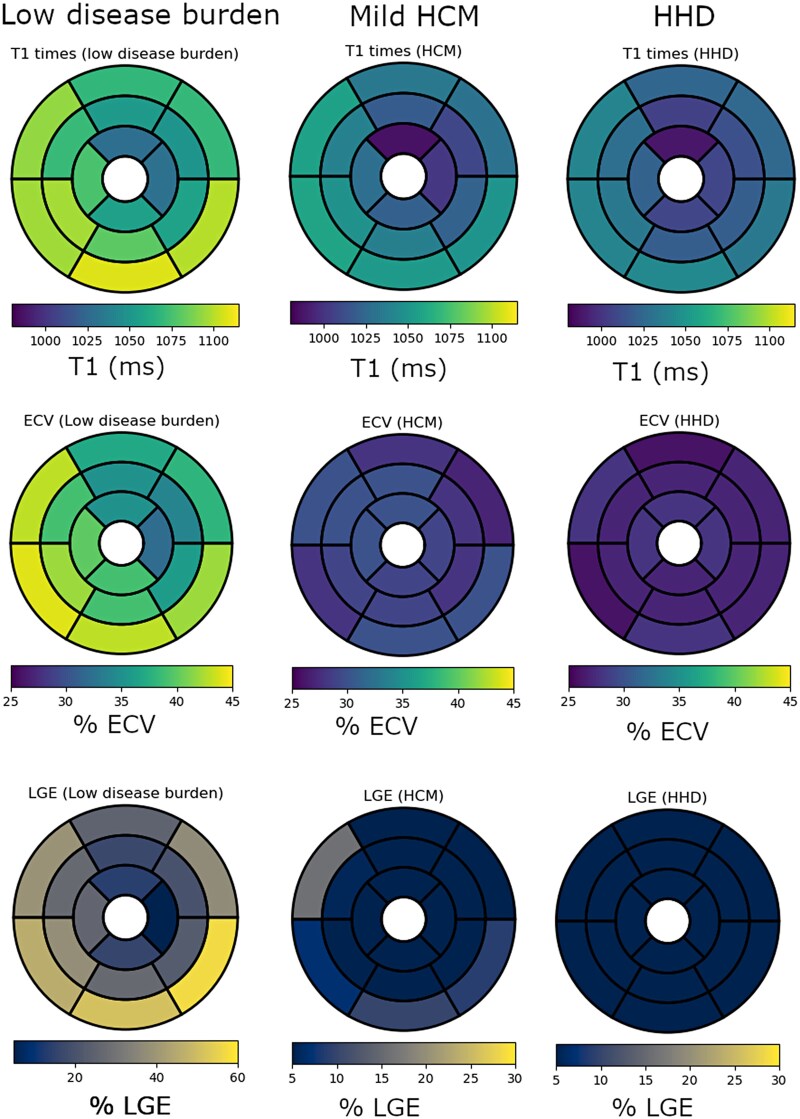
Segmental analysis of tissue characterization of low burden ATTR CA, mild HCM and HHD. Color map representation of mean T1 times, ECV, and LGE% segmental values on a 16-segment AHA model, in low burden ATTR compared to mild HCM and HHD. Normal reference values for T1 = 1000 ± 18 ms, ECV =28 ± 1.5% and LGE 0%.

Global LV T1 times, ECV, and LGE % were significantly higher in patients with low burden ATTR CA compared to mild HCM and HHD, mean global T1 1069 ± 38 ms vs. 1028 ± 31 ms and 1014 ± 27 ms, respectively, *P* < 0.001; mean global ECV 39 ± 4% vs. 29 ± 3% and 27 ± 2%, respectively, *P* < 0.001; mean %LGE 29 ± 15% vs. 4 ± 3% and 1.3 ± 2%, respectively, *P* < 0.001 (*Table 3*). Among low burden ATTR CA, abnormal CMR tissue characterization was more prevalent in the basal inferolateral and inferior segments compared to HCM, which predominantly involved the basal anteroseptal segment (*Figure 5*).

Left atrial ejection fraction (LAEF) was significantly lower among low burden ATTR, mean LAEF 34 ± 14% vs. 53 ± 11% in mild HCM and 54 ± 14% in HHD, *P* = 0.001. Atrial wall and RV wall LGE were more frequently encountered in low burden ATTR compared to mild HCM and HHD (*Table 3*).

**Figure 6:**
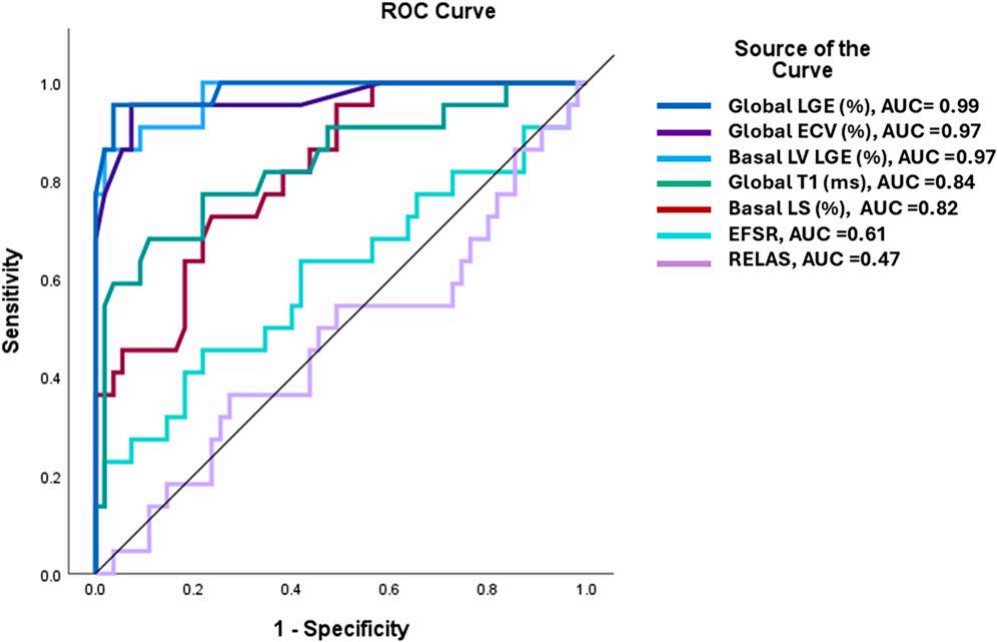
Combined receiver operator characteristic curves of CMR parameters for differentiating low burden ATTR cardiac amyloidosis from mild HCM and HHD.

ROC analysis of CMR parameters differentiating low burden ATTR CA from mild HCM and HHD found an area under the curve (AUC) of 0.99 (95% CI 0.96–1.01) for global LV LGE%, AUC 0.97 (95% CI 0.94–1.01) for mean % LV LGE in the basal segments, AUC 0.97 (95% CI 0.92–1.01) for global ECV and AUC 0.84 (95% CI 0.73–0.95) for global T1 times, AUC of 0.82 (95% CI 0.72–0.92) for basal LS, AUC of 0.61 (95% CI 0.45–0.76) for EFSR and AUC 0.47 (95% CI 0.32–0.62) for RELAS (*Figure 6*). The following CMR metric cut-offs had high diagnostic accuracy for distinguishing low burden ATTR from mild HCM and HHD, global ECV 32% (sensitivity of 95%, specificity 93%), global T1 1054 ms (sensitivity 68%, specificity 89%), global LV LGE 11% (sensitivity 95%, specificity 96%) and basal segment LV LGE 22% (sensitivity 86%, specificity 98%).

## Discussion

Our study demonstrated distinctive clinical and imaging phenotypes in patients with low burden disease on CMR (myocardial ECV ≤43%). They had mildly increased serum cardiac biomarkers, mild septal wall thickening, and significant basal segment involvement on CMR-derived tissue characterization, and strain analyses. In our study, patients with low disease burden (ECV ≤43%) had more favourable outcomes compared to those with ECV >43%. The extent of amyloid infiltration on CMR tissue characterization (%LGE, T1, and ECV) had excellent discriminatory performance in distinguishing low burden ATTR CA from mild HCM and HHD (*Graphical Abstract*).

Typical CMR findings of ATTR CA include LV wall thickening, significant elevation in myocardial ECV and T1 times, and diffuse subendocardial or transmural LV enhancement with or without enhancement of the RV and atria.^3,5,18^ Our findings show that this classic depiction of CMR findings in ATTR CA may not be appreciated in patients with low burden disease and are more representative of moderate or high disease burden.

When we compared the tissue parametric mapping and LV LGE % measures in low burden ATTR CA with potential disease mimickers, we found myocardial involvement predominantly affected the basal inferior and inferolateral segments, whereas mild HCM was characterized by involvement of the basal anteroseptal segment, and HHD had minimal myocardial fibrosis. Furthermore, tissue mapping and LGE appear to outperform traditional strain-based measures including RELAS and EFSR for distinguishing low burden ATTR from potential mimickers (*Figure 6*).

We have shown that the extent of cardiac infiltration on CMR as characterized by myocardial LGE, global T1, and ECV may help distinguish low burden ATTR CA from mild HCM or HHD. These findings should be confirmed in larger ATTR CA cohorts before being applied clinically. Nevertheless, this study highlights the potential role of CMR to identify low-burden ATTR CA cases, which are more challenging to diagnose in clinical practice. This could lead to the earlier identification of ATTR CA targeting therapies and a greater potential to impact disease trajectory.

In patients with low burden ATTR CA, myocardial LGE was seen predominantly in the basal segments, especially inferior and inferolaterally (*Figure 2*). Previous reports involving CMR LGE imaging have demonstrated a predilection for basal segment involvement in population studies and various other cardiac disease processes, including myocarditis, sarcoidosis, Anderson-Fabry disease, and neuromuscular dystrophies.^19–21^ Mechanisms to explain this preferential involvement of the basal segments have not been fully elucidated, but we hypothesize that this may relate to differences in the regional expression of heparan sulfate proteoglycans. These proteins are commonly found in the myocardial extracellular matrix, and their production increases in response to mechanical stress.^22^ Importantly, these proteins have been associated with amyloid deposition and pathogenicity.^23^ Cardiac magnetic resonance-based studies of healthy individuals have shown that wall stress is higher in the LV basal segments relative to the apex and that the basal inferior and inferolateral segments have the highest regional strain values.^24,25^ Therefore, regional variation in LV mechanical forces and resulting changes to the myocardial extracellular matrix could explain this non-uniform pattern of cardiac amyloidosis deposition.

It is not clear whether low burden ATTR CA represents an early disease stage or patients with slower disease progression. The latter possibility highlights the potential role of innate amyloidosis homeostatic mechanisms beyond disease-modifying therapies. For example, the impact of estrogen in terms of reducing the detrimental effects of amyloid fibrils on cardiac function has been previously described, suggesting a potential protective effect of this hormone.^26^ This would be consistent with the observation that women with cardiac amyloidosis have less severe morphological and functional involvement compared to men, with these differences less pronounced in post-menopausal women.^27^ In our study, we noted a relatively higher proportion of females in the low burden group compared to patients with moderate or high burden; however, this sex difference did not reach statistical significance.

### Limitations

Despite the novel findings of our study, there are several potential limitations. The retrospective inclusion of patients from a single tertiary care centre may result in selection bias. The sample size of our low burden ATTR CA cohort was small and could lead to under-sampling errors, overfitting in the ROC analyses, and limit subgroup analyses, including sex differences. Nevertheless, the CMR phenotype of low disease burden ATTR CA group was clearly distinctive from the higher burden groups and hypertrophic phenocopies. The results of this study, including the differences in phenotype and outcome between low and higher burden ATTR CA and the cut-offs identified in the ROC analysis, should be externally validated in larger cohorts. Nevertheless, our findings are novel and provide a potential framework for investigating patients with suspected low burden ATTR CA. The small cohort size and relatively short follow-up period impacts survival analysis results. However, previous studies of CMR-derived myocardial ECV confirm an association with overall survival in patients with higher burden ATTR CA.^8^

## Conclusion

In patients with ATTR CA referred for CMR, low burden phenotype is characterized by normal or mildly increased LV wall thickness with abnormal tissue characterization and strain, particularly in the basal segments. Tissue characterization features such as global LGE and ECV appear to differentiate low burden ATTR CA from patients with similar disease phenotypes, including HCM and HHD.

### Clinical perspectives

- Our study illustrates the potential role of CMR-derived ECV for staging ATTR CA.
- Patients with low burden ATTR CA have a characteristic pattern of myocardial infiltration in the basal left ventricular segments and relative sparing of the mid and apical regions that is distinctive from moderate and high burden ATTR CA.
- Tissue characterization imaging (myocardial ECV and LGE) can be used to distinguish low burden ATTR CA from potential mimickers (mild HCM and HHD) and appears to outperform traditional strain-based measures. These features could facilitate the early detection of ATTR CA in CMR studies performed for the work-up of undifferentiated LV hypertrophy.

## Supplementary Material

qyag038_Supplementary_Data

## Data Availability

The authors are willing to share study data upon reasonable request and according to institutional regulations.

## Lead author biography

Dr Mengesha earned her medical degree from Jimma University in Ethiopia, and completed residency training in internal medicine, followed by cardiology specialization at Shamir medical centre in Israel. She then pursued advanced imaging fellowship training, including cardiac CT, cardiac MRI, and nuclear cardiology, at the University of Ottawa Heart Institute, where she attained APCA board certification in all three imaging modalities. She is currently undertaking additional subspecialty training in structural heart imaging at St. Paul’s Hospital in Vancouver.

Throughout her medical education and residency, she was recognized for her leadership skills and dedication to mentoring medical students. During her cardiology training, she secured a competitive research grant, a work that was later presented at the European Society of Cardiology (ESC) Congress. Her research during her fellowship focuses on the role of multimodality imaging in the diagnosis and risk stratification of cardiomyopathies and structural heart disease. She has presented her work at major international scientific meetings, including the American College of Cardiology (ACC), American Society of Nuclear Cardiology (ASNC), Society for Cardiovascular Magnetic Resonance (SCMR), and Society of Cardiovascular Computed Tomography (SCCT). In 2025, she received recognition at the Ottawa Cardiovascular Research Day for her abstract presentation and received a travel bursary research award by the Canadian Society of Echocardiography. She was also selected for the American Society of Nuclear Cardiology’s Leadership Development Program (LDP), a distinguished initiative designed to foster future leaders in the field.
