# Supplementary material for: Low burden transthyretin cardiac amyloidosis on cardiac magnetic resonance: comprehensive phenotyping and distinction from hypertrophic phenocopies
Source: Eur Heart J Imaging Methods Pract. 2026 Feb 28;4(1):qyag038. doi: 10.1093/ehjimp/qyag038 (PMC12993924; doi:10.1093/ehjimp/qyag038)
Supplement: qyag038_Supplementary_Data [file qyag038_supplementary_data.docx]

**Appendix**

Supplemental Methods: Page 2

Supplemental Results: Page 3

References: Page 4

Supplemental Figure 1: Page 5

Supplemental Figure 2: Page 6

Supplemental Figure 3: Page 7

Supplemental Figures Legends: Page 8

Supplemental Table 1: Pages 9-10

Supplemental Table 2: Pages 11-12

Supplemental Table 3: Page 13

**Supplemental Methods - CMR protocol**

Cardiac magnetic resonance (CMR) examinations were performed on a 1.5 T scanner (Magnetom Aera, Siemens Healthineers, Erlangen, Germany) with image acquisition according to recommended protocols for patients with non-ischemic cardiomyopathy [1]. Breath-hold cine images were acquired in four chamber long axis, short axis oblique, two chamber long axis, and three chamber orientations using a segmented balanced steady-state free precession (SSFP). In all cases, cine images were acquired with 8 mm thick slices, 2 mm gap, and 25 cardiac phases. For SSFP readout cine images, the scan parameters were as follows: temporal resolution, 39 ms; echo time (TE) 1.04 ms; 153-163 phase encode lines; flip angle 54°. The field of view and acquisition matrix was 319 mm x 399 mm and 240 x 192, respectively for SAO cines; 270 mm x 360 mm and 180 x 240 for four chamber cines; 270 mm x 360 mm and 153 x 240 for three chamber cines; 299 mm x 399 mm and 240 x 180 for two chamber cines. T1 maps and late gadolinium enhancement (LGE) images were acquired in the same position and orientation as cine images. Native and post contrast T1 maps were acquired using a balanced SSFP readout modified Look-Locker inversion recovery technique (native (5(3)3) and post-contrast (4(1)3(1)2), 8 mm slice thickness). Native and post contrast T1 maps were acquired in 3 mid ventricular short axis oblique views as well as 3- and 4-chamber views. LGE imaging was acquired using a breath-hold 2D segmented phase sensitive inversion recovery spoiled gradient echo sequence approximately 8-10 min after administering 0.15 mmol/kg of gadolinium-based contrast (Gadovist, Bayer Healthcare, Berlin, Germany). The optimal inversion time was selected with the aid of an inversion time (TI) scout sequence. For LGE images, the scan parameters were as follows: repetition time 3.8 ms; echo time 2.7 ms; flip angle 25°. Image analysis for cardiac chamber sizes, function, left ventricular (LV) mass and parametric mapping was performed using a semiautomated segmentation software (CVI42 Version 6.1, Circle Inc, Calgary, AB, Canada), following guidelines for post-processing [2].

The endocardial LV borders corresponding to the end diastolic and end systolic phases of each cine series were traced semiautomatically and manually corrected by an experienced reader. The papillary muscles and trabeculations were included in the LV volume. LV and right ventricular (RV) volume and LV mass were indexed to body surface area. LV global and segmental radial (GRS), circumferential (GCS) and longitudinal strain (GLS) were measured by feature tracking. GLS was obtained from strain analysis based on 2 -chamber, 3-chamber and 4-chamber cine images. GRS and GCS were obtained from short-axis oblique cine images after excluding basal slices containing the left ventricular outflow tract. Strain analysis was not performed in patients with limited cine image quality due to significant cardiac motion artifacts. The segmental longitudinal strain values were not included in the final analysis due to frequent spurious measurements. Post-processing and analysis of T1 maps were performed by an experienced CMR reader (BM). ECV was derived from pre-contrast and post contrast T1 times as well as hematocrit value calculated using the formula ECV = (1 - hematocrit) × (ΔR1myocardium/ΔR1blood) [2].

**Supplemental Results**

**ECG and cardiac biomarkers**

ECG data was available in 82 patients, 33% of which were in atrial fibrillation and 8% were in atrial flutter. First degree atrioventricular block was seen in 13% of patients. Right bundle branch block and left bundle branch block were noted in 19% and 11% of the cases, respectively. Low voltage criteria were met in 42% of the study population, and 45% of the patients had a pseudo infarct pattern. Regarding cardiac biomarkers, median NT pro BNP and median high sensitivity troponin T were 2082 (IQR 886-4629) and 50ng/L, (IQR 36-76) respectively.

**Multimodality imaging findings by disease burden**

Data on echocardiography was available on 96% of patients with transthyretin cardiac amyloidosis (ATTR CA). Left ventricular ejection fraction (LVEF) was significantly reduced in the high burden cohort compared to the low burden cohort (mean LVEF 36%±14, vs. 52%±12, p<0.001). Left ventricular mass was associated with disease burden (mean LV mass index 71 ± 16 g/m^2^ in low burden, 81±17 g/m^2^ in moderate and 99±16 g/m^2^ in high burden cohort, p <0.001).

Among 80 (96%) patients that underwent pyrophosphate imaging, all had grade 2-3 radiotracer uptake in the myocardium. There was no significant difference in the Perugini score however the heart to contralateral lung ratio (H/CL ratio) increased in stepwise fashion according to disease burden (1.7±0.2 in low disease burden, 1.7±0.3 in moderate and 1.89±0.2 in high, p=0.028).

**Inter-Reader Agreement**

The intraclass correlation coefficients (ICC) between the two readers for global myocardial T1 and ECV were 0.99, (95% confidence interval (CI) 0.97-0.99) and 0.96 (95% CI 0.92-0.98) respectively. The ICC for segmental myocardial T1 were 0.99 (95% CI 0.98-0.99), 0.98 (95% CI 0.95-0.99) and 0.99 (95% CI 0.98-0.99) for the basal, mid and apical LV segments, respectively. The ICC for myocardial ECV were 0.93 (95% CI 0.87-0.96), 0.90 (95% CI 0.82- 0.95) and 0.86 (95% CI 0.74- 0.93) for the basal, mid-LV, and apical segments, respectively, indicating good to excellent agreement.

**Strain analysis**

Among patients with ATTR CA, segmental strain analysis showed abnormal strain pattern preferentially in the basal septum (Supplemental Figure 3). Furthermore, the degree of strain abnormality was more pronounced with low burden ATTR compared to mild HCM and HHD (Supplemental Figure 3).

**References**

1. Kramer CM, Barkhausen J, Bucciarelli-Ducci C et al. Standardized cardiovascular magnetic resonance imaging (CMR) protocols: 2020 update. J Cardiovasc Magn Reson. 2020 Feb 24;22(1):17. doi: 10.1186/s12968-020-00607-1. PMID: 32089132; PMCID: PMC7038611.
2. Schulz-Menger J, Bluemke DA, Bremerich J et al. Standardized image interpretation and post-processing in cardiovascular magnetic resonance - 2020 update: Society for Cardiovascular Magnetic Resonance (SCMR): Board of Trustees Task Force on Standardized Post-Processing. J Cardiovasc Magn Reson. 2020 Mar 12;22(1):19. doi: 10.1186/s12968-020-00610-6. PMID: 32160925; PMCID: PMC7066763.
3. Yang, Wenjing et al. “Myocardial Strain Measurements Derived From MR Feature-Tracking: Influence of Sex, Age, Field Strength, and Vendor.” *JACC. Cardiovascular imaging* vol. 17,4 (2024): 364-379. doi:10.1016/j.jcmg.2023.05.019

**Supplemental Figure 1**


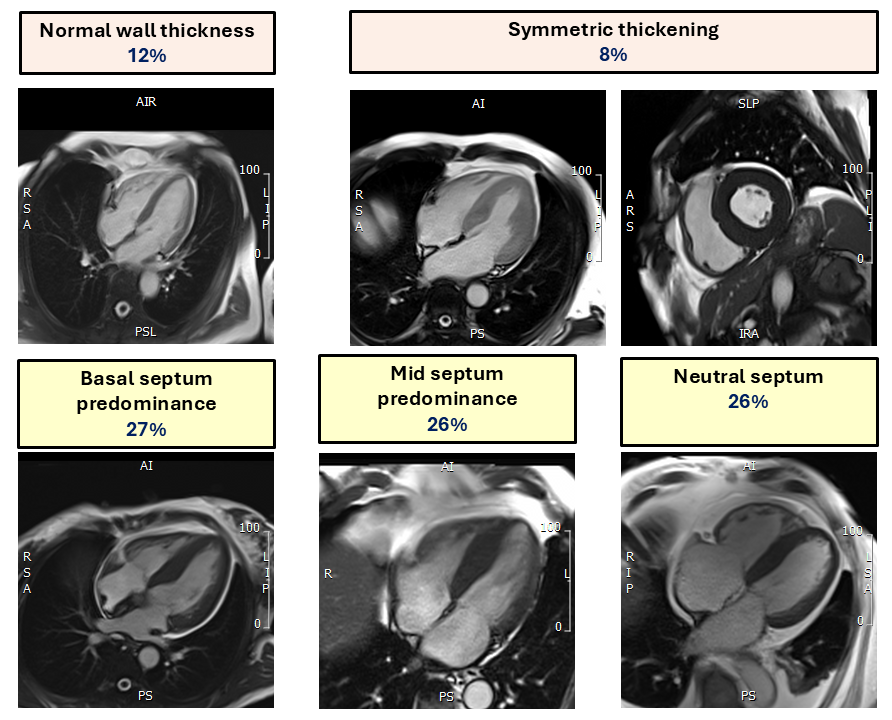


**Supplemental Figure 2**


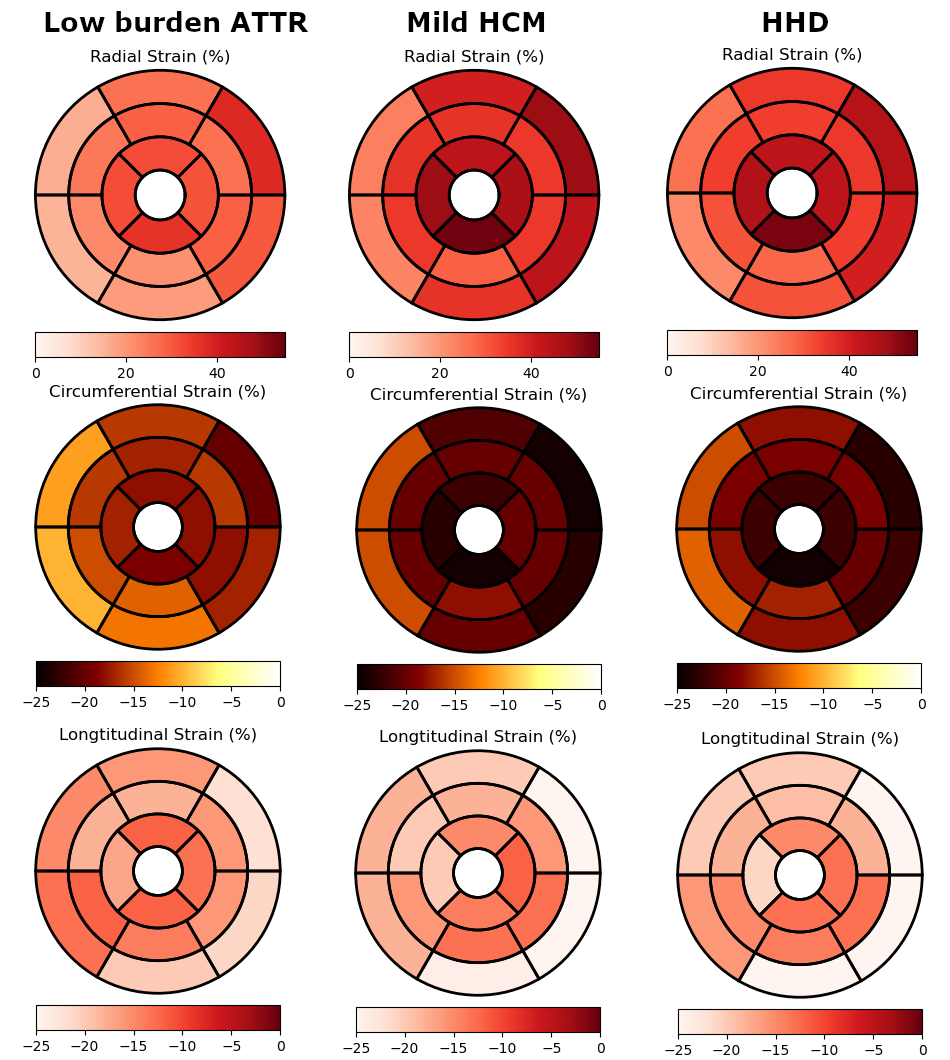


**Supplemental Figure 3**


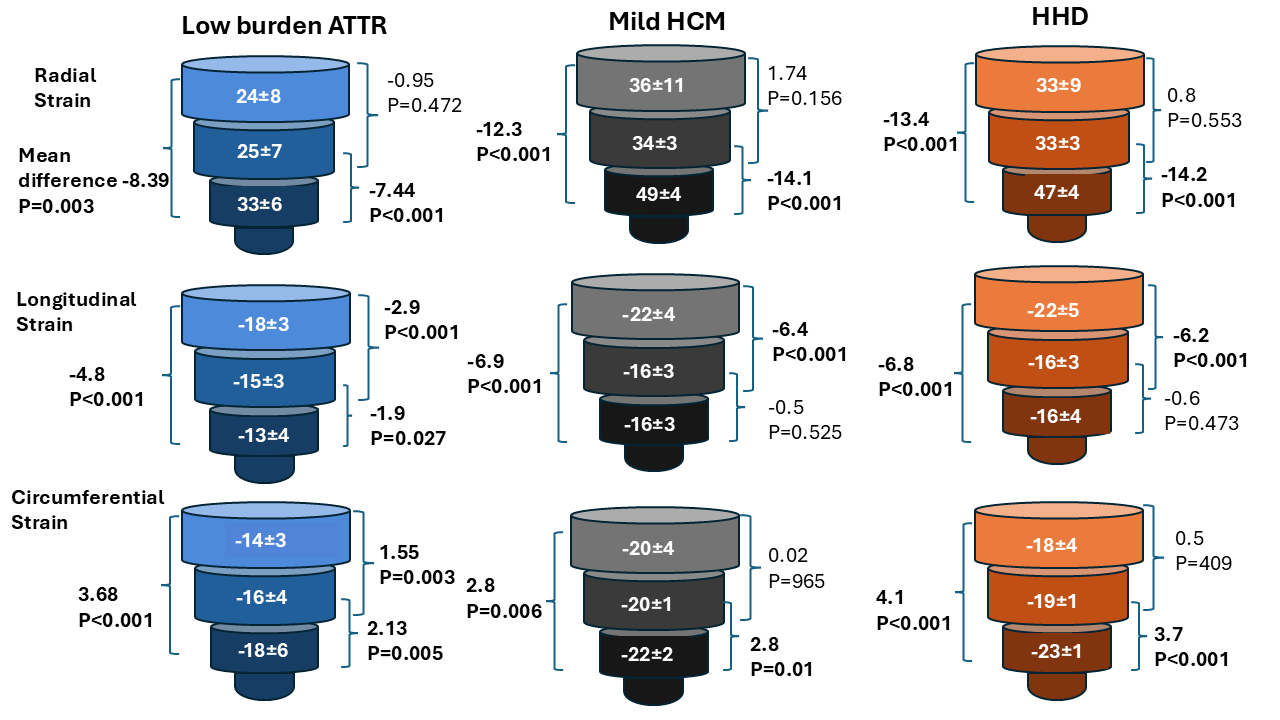


**Supplemental Figure Legends**

**Supplemental Figure 1:** Patterns of LV wall thickening on CMR in ATTR CA.

Most patients (80%) with ATTR showed asymmetric LV wall thickening predominantly in the basal and mid septum or with neutral septal thickening. 12% had normal wall thickness and symmetric LV wall thickening was seen in 8% of patients.

**Supplemental Figure 2:** Segmental analysis of the radial, circumferential and longitudinal strain in low burden ATTR CA compared to mild HCM and HHD. Low burden ATTR CA was characterized by lower strain values in the basal and mid-septal and inferior segments compared to mild HCM and HHD.

**Supplemental Figure 3:** Basal to apical gradient in radial, longitudinal and circumferential strain in low burden ATTR CA, mild HCM and HHD. Reference normal values LV global longitudinal strain, LV global radial strain, and LV global circumferential strain are -18.4% (95% CI: -19.2% to -17.6%), 43.7% (95% CI: 40.0%-47.4%), and -21.4% (95% CI: -22.3% to -20.6%) respectively [3].

**Supplemental Tables**

**Supplemental Table 1**: Baseline characteristics according to Gillmore stage

| **Clinical variables** | **Stage 1**  **N = 45** | **Stage 2**  **N = 28** | **Stage 3**  **N = 9** | **P value** |
| --- | --- | --- | --- | --- |
| **Age** | 76±10 | 81±7* | 84±4† | **<0.001** |
| **Male sex** | 39 (87) | 25 (89) | 8 (89) | 0.941 |
| **Variant ATTR** | 6 (13) | 1 (4) | 1 (11) | 0.381 |
| **Body mass index (kg/m^2^)** | 26±4 | 25±4 | 27±3 | 0.587 |
| **Systolic BP (mmHg)** | 132±19 | 122±17* | 120±9 | **0.020** |
| **Diastolic BP (mmHg)** | 79±12 | 73±17 | 66±7† | **0.003** |
| **Heart rate (/min)** | 76±14 | 71±17 | 64±24 | 0.233 |
| **Hypertension** | 24 (53) | 18 (64) | 9 (100) | **0.030** |
| **Diabetes mellitus** | 10 (22) | 2 (7) | 1 (11) | 0.211 |
| **Atrial fibrillation** | 16 (36) | 18 (64) | 5 (56) | 0.051 |
| **Cerebrovascular disease** | 5 (11) | 5 (18) | 0 | 0.343 |
| **Coronary heart disease** | 13 (29) | 6 (21) | 2 (22) | 0.754 |
| **Congestive heart failure**  **HFrEF**  **HFmrEF**  **HFpEF** | 29 (64)  7 (24)  15 (52)  7 (24) | 20 (71)  8 (40)  9 (45)  3 (15) | 9 (100)  2 (22)  6 (67)  1 (11) | 0.101 |
| **NYHA class**  **I**  **II**  **III/IV** | 18 (40)  17 (38)  10 (22) | 6 (21)  13 (46)  9 (32) | 2 (22)  4 (44)  3 (33) | 0.511 |
|  |  |  |  |  |
| **Diuretics** | 18 (40) | 19 (68)* | 7 (67)† | **0.021** |
| **Furosemide dose (mg)** | 0 (0-20) | 30 (0-80) * | 40 (40-80)**†** | **<0.001** |
| **Tafamidis** | 28 (62) | 19 (68) | 6 (67) | 0.879 |
| **Low voltage** | 16 (36) | 10 (36) | 4 (44) | 0.894 |
| **Pseudo-infarct pattern** | 19 (42) | 12 (43) | 4 (44) | 0.993 |
| **NT-pro BNP (ng/L)** | 1134 (521-1706) | 4160 (3124-6490)* | 7361 (5306-8501) †⁋ | **<0.001** |
| **Troponin T (ng/L)** | 38 (27-56) | 66 (46-105)* | 96 (78-136) † | **<0.001** |
| **eGFR (ml/m/1.73m sq)** | 76 (66-84) | 58 (46-66)* | 33 (27-41) †⁋ | **<0.001** |
| * P<0.05 between Stage 1 and 2 groups  **†** P<0.05 between Stage 1 and 3 groups  ⁋ P<0.05 between Stage 2 and 3 groups  Continuous data is provided as mean ± standard deviation or median and interquartile ranges and categorical data is provided as counts and percentage.  Abbreviations: ATTR = transthyretin amyloidosis, HFrEF= heart failure with reduced ejection fraction, HFmrEF= heart failure with mildly reduced ejection fraction, HFpEF= heart failure with preserved ejection fraction, NYHA class= New York Heart Association class, BP = blood pressure, ACE= angiotensin converting enzyme, SGLT2I= Sodium glucose cotransporter II inhibitor, NT-BNP = N terminal B-type natriuretic peptide, eGFR= estimated glomerular filtration rate (estimated using CKD-EPI formula) | | | | |

**Supplemental Table 2:** CMR findings according to Gillmore stage

| **Imaging parameters** | **Stage 1**  **n=46** | **Stage 2**  **n=28** | **Stage 3**  **n=9** | **P value** |
| --- | --- | --- | --- | --- |
| **LVEF (%)** | 49±11 | 44±16 | 44±13 | 0.300 |
| **LVEDVi (ml/m^2^)** | 82±18 | 92±21 | 85±22 | 0.168 |
| **LVESVi (ml/m^2^)** | 38 (31-50) | 47 (37-58) | 44 (33-62) | 0.096 |
| **Maximal wall thickness (mm)** | 16 ±3 | 16 ±3 | 16 ±3.4 | 0.696 |
| **LV mass index (g/m2)** | 80±19 | 86 ±19 | 92 ±20 | 0.174 |
| **RVEF (%)** | 49 ±11 | 44 ±14 | 43 ±12 | 0.217 |
| **RVEDVi (ml/m^2^)** | 79 ±17 | 95 ±21* | 86 ± 23 | **0.017** |
| **RVESVi (ml/m^2^)** | 38 (33-47) | 52 (36-70)* | 40 (34-61) | **0.030** |
| **LAVi (ml/m^2^)** | 57±10 | 67 ±13 | 61 ±10 | 0.054 |
| **RAVi (ml/m^2^)** | 56 ±23 | 64 ±26 | 62 ±29 | 0.434 |
| **Global Longitudinal Strain (%)** | -10 ±5 | -10 ±3 | -9 ±3 | 0.303 |
| **Global Radial Strain (%)** | 21 ±22 | 17 ±6 | 17 ± 6 | 0.053 |
| **Global Circumferential Strain (%)** | -14 ±4 | -11 ±6 | -9 ±8 | 0.051 |
| **Global T1 (ms)** | 1093 ±51 | 1114 ±39 | 1108 ±47 | 0.160 |
| **Global ECV (%)** | 49 ±11 | 53 ± 9 | 53 ± 8 | 0.225 |
| **LGE % of LV mass** | 39 ±18 | 44 ±15 | 43 ±21 | 0.389 |
| ***** P<0.05 between stage 1 and 2 groups  **†** P<0.05 between stage 1 and 3 groups  **⁋** P<0.05 between stage 2 and 3 groups  Continuous data is provided as mean ± standard deviation or median and interquartile ranges and categorical data is provided as counts and percentage.  Abbreviations: LV = left ventricular, RV = right ventricular, EF = ejection fraction, EDVi = end diastolic volume index, ESVi = end systolic volume index, LAVi = left atrial volume index, RAVi = right atrial volume index, LGE = late gadolinium enhancement. N.B. index = indexed to body surface area. | | | | |

**Supplemental Table 3**: Baseline characteristics of low burden ATTR cardiac amyloidosis, mild hypertrophic cardiomyopathy and hypertensive heart disease groups

| **Clinical variables** | **Low burden ATTR CA**  **n=22** | **Mild HCM**  **n=27** | **HHD**  **n=28** | **P value** |
| --- | --- | --- | --- | --- |
| **Age** | 76±9 | 61±12* | 63±14† | **<0.001** |
| **Male sex** | 16 (73%) | 13 (48%) | 14 (50%) | 0.167 |
| **Body mass index (kg/m^2^)** | 24.8 (23-29) | 25.6 (23-29) | 27.9 (25-32) | 0.054 |
| **Hypertension** | 16 (73%) | 0* | 28 (100%)† | **<0.001** |
| **Dyslipidemia** | 13 (59%) | 6 (22%)* | 10 (36%) | **0.029** |
| **Diabetes mellitus** | 4 (18%) | 0 | 3 (11%) | 0.083 |
| **Atrial fibrillation** | 6 (27%) | 1 (3.7%)* | 3 (11%) | **0.046** |
| **Coronary artery disease** | 6 (27%) | 0* | 2 (7.4%) | **0.006** |
| **Congestive heart failure** | 14 (65%) | 1 (3.7%)* | 1 (3.6%)† | **<0.001** |
| **Aspirin** | 11 (50%) | 4 (14.8%)* | 7 (25%) | **0.022** |
| **Statin** | 15 (68%) | 16 (59.3%) | 12 (43%) | 0.183 |
| **Beta Blockers** | 9 (41%) | 11 (41%) | 10 (36%) | 0.907 |
| **Calcium Channel Blockers** | 4 (18%) | 1 (4%) | 13 (46%)⁋ | **<0.001** |
| **ACE inhibitors** | 5 (23%) | 1 (3.7%) | 10 (36%)⁋ | **0.013** |
| **Diuretics** | 8 (36%) | 1 (3.7%)* | 4 (14%) | **0.009** |
| ***** P<0.05 between low burden ATTR CA and mild HCM cohorts  **†** P<0.05 between low burden ATTR CA and HHD cohorts  **⁋** P<0.05 between mild HCM and HHD cohorts  Continuous data is provided as mean ± standard deviation or median and interquartile ranges and categorical data is provided as counts and percentage.  Abbreviations: ATTR CA = transthyretin cardiac amyloidosis, HCM = hypertrophic cardiomyopathy, HHD = hypertensive heart disease, ACE = Angiotensin converting enzyme | | | | |
